# Role of various complete blood count parameters in predicting the success of single-dose Methotrexate in treating ectopic pregnancy

**DOI:** 10.12669/pjms.345.15356

**Published:** 2018

**Authors:** Ahkam Goksel Kanmaz, Abdurrahman Hamdi Inan, Emrah Beyan, Adnan Budak

**Affiliations:** 1 Dr. Ahkam Goksel Kanmaz, Department of Obstetrics and Gynecology, Tepecik Training and Research Hospital, Izmir, Turkey; 2 Dr. Abdurrahman Hamdi Inan, Department of Obstetrics and Gynecology, Tepecik Training and Research Hospital, Izmir, Turkey; 3 Dr. Emrah Beyan, Department of Obstetrics and Gynecology, Tepecik Training and Research Hospital, Izmir, Turkey; 4 Dr. Adnan Budak, Izmir Provincial Health Directorate, Izmir, Turkey

**Keywords:** Ectopic pregnancy, Methotrexate, Neutrophil-to-lymphocyte ratio

## Abstract

**Objective:**

The protocol of 15% BhCG decrease between Days four and seven is frequently used for evaluating the success of methotrexate administration in treating ectopic pregnancy. Our objective was to study the usage of hematologic parameters for evaluating the success of methotrexate administration in treating ectopic pregnancy.

**Method:**

This study was conducted between February 2014 and December 2016. Data of 434 patients were retrospectively scanned for the study. One hundred sixty-one patients whose Day one, four and seven results were recorded in the hospital information system and who were followed up until their BhCG levels decreased <10 IU/L were enrolled in the study. Three types of complete blood cell count parameters of the patients were used: 1) Neutrophil-to-lymphocyte ratio (NLR). 2) Platelet distribution width (PDW), 3) Platelet count (PLT).

**Results:**

Patients were separated into two groups as those who were treated with single-dose methotrexate and those who required surgical treatment. A significant difference was detected between the groups in terms of NLR levels on Days 1, 4, and 7 (p=0.012, p=0.035, and p=0.001, respectively). There was no significant difference detected between the groups for PDW and PLT counts on Days one, four and seven.

**Conclusions:**

NLR can also be used as an alternative to BhCG for evaluating the success of single-dose methotrexate administration in treating ectopic pregnancy. However, there is need for further studies on this topic.

## INTRODUCTION

Ectopic pregnancy constitutes approximately 1%-2% of all pregnancies and is observed in 6%–16% of all patients who are admitted to emergency services with vaginal bleeding and inguinal pain.^1^,^2^ A decline in morbidity and mortality rates associated with ectopic pregnancy has been reported following developments in imaging methods and the definition of the BhCG monitoring protocol.^1^

Because of more frequent diagnosis of ectopic pregnancies, the use of medical agents for treatment has increased as an alternative to surgical treatment because they are more effective, cost-effective, and safer. The use of methotrexate in the medical treatment of ectopic pregnancy was defined by Stovval TG et al.^3^ in 1989. Although the response rate to single-dose methotrexate administration is between 65%and 95%, there is need for repeated methotrexate administration at a rate of 3%–27%.^4^,^5^

The most commonly used protocol for evaluating the success of methotrexate administration in treating ectopic pregnancy is the protocol with 15% BhCG decrease between Days 4 and 7, which was defined by Kirk E et al.^6^ In addition to monitoring BhCG levels, levels of many markers, such as creatine kinase, myoglobulin, progesterone, relaxin, cancer antigen 125 (ca 125), metalloprotease-12, and pregnancy-associated plasma protein A (PAPP-A), were studied for ectopic pregnancy monitoring and treatment success.^7^,^8^

In the study conducted by Turgut et al., it was observed that there is an increase in MPV and leukocyte counts during ectopic pregnancy.^9^ Certain studies on the inflammatory process during ectopic pregnancy have revealed an increase in the levels of markers, such as platelet count (PLT), platelet distribution width (PDW), and neutrophil-to-lymphocyte ratio (NLR); in the recent years, these have been particularly recommended for the follow-up of treatment and for determining surgical requirement during ectopic pregnancy.^10^-^13^

In this study, our objective was to study the usage of recommended hematologic parameters for evaluating the success of single-dose methotrexate administration treating ectopic pregnancy.

## METHODS

Our study is a retrospective analysis of patients who were admitted to Tepecik Training and Research Hospital, Department of Obstetrics and Gynecology, who were diagnosed with tubal ectopic pregnancy, and received treatment between February 2014 and December 2016. Approval was acquired from the ethics committee of Tepecik Training and Research Hospital.

Our clinical approach included single-dose methotrexate administration, which is the primary medical treatment method, to patients diagnosed with tubal ectopic pregnancy with stable hemodynamics, no contraindications for methotrexate administration, BhCG <6000 IU/L at the time of diagnosis, and ectopic pregnancy mass size < 40 mm. On the contrary, surgical treatment was performed on patients who are diagnosed with ectopic pregnancy and did not meet these criteria, i.e., whose hemodynamics deteriorated because reasons such as tubal rupture during the follow-up, who had <15% BhCG decrease between Days four and seven following single-dose methotrexate administration, and who were not responsive to the medical treatment.

Patients who were diagnosed with ectopic pregnancy and considered to be suitable for methotrexate treatment in our clinic and had complete blood cell count after methotrexate admission on Days one, four and seven, followed up in our hospital until their BhCG level decreased <10 IU/L following the treatment were enrolled into the study.

The treatment was considered to be successful if the patients’ BhCG level decreased<10 IU/L after only single-dose methotrexate treatment, whereas single-dose methotrexate treatment of patients who underwent surgery was regarded to be unsuccessful. According to these criteria, patients were separated into two groups as those who had success with single-dose methotrexate treatment and those who required surgery. NLR, PDW, and PLT levels on Days 1, 4, and 7 were compared between the groups.

### Statistical analysis

Statistical analysis was performed using SPSS 22.0 software. Normality tests of variables were conducted based on the amount of data. Normal distribution was rejected for values <0.05, whereas it was accepted otherwise. Although independent t-test was performed for continuous parametric variables, Mann–Whitney U test was performed for non-parametric variables and chi square test was performed for categorical variables. P <0.05 was considered to be significant. As additional analyses for complete blood count parameters were found to be associated with single-dose methotrexate, roc curve, sensitivity (sen), specificity (spe), positive predictive value, and negative predictive value were calculated with 95% CI.

## RESULTS

Data of 434 patients who were diagnosed with tubal ectopic pregnancy and treated with single-dose methotrexate in our clinic were scanned. Two hundred and seventy-three patients whose complete blood count parameters on Days 1, 4, and 7 were not available in the hospital information system and whose BhCG values were detected to be >6000 IU/L were excluded from the study, and in total, 161 patients were included in the study. One hundred and thirty-four out of 161 patients was successfully treated with single-dose methotrexate, whereas 27 patients needed surgery. The patients were separated into two groups based on the treatment received. There was no statistically significant difference detected between the groups in terms of patient age, parity, tubal ectopic pregnancy side, and size of the mass measured using transvaginal ultrasound (Table-I). NLR on Days one, four and seven of the patient group with successful treatment with single-dose methotrexate were found to be statistically significantly lower compared with that of the patient group requiring surgery (Table-II). PLT and PDW used for evaluating the success of single-dose methotrexate treatment were found to be lower in the group that did not require surgery, but no statistical significance was detected (Table-II).

**Table-I:** Demographic and clinical details.

	Successful single-dose treatment (n = 134)	Surgical treatment (n = 27)	P-value
Age (mean ± s.d.)	31.01 ± 5.701	32 ± 5.174	0.500^a^
Parity (n,%)			0.615^a^
0	52 (39.7%)	11 (40.7%)	
≥1	82 (60.3%)	16 (59.3%)	
***Adnexal mass average size, mm*** (mean±s.d.)	21.661 ± 8.2374	24.811 ±7.9234	0.551^a^
***Ectopic pregnancy side***			
Right	67 (82.7%)	14 (17.3%)	0.514^b^
Left	67 (83.8%)	13 (16.2%)	

a independent sample t-test, (mean±s.d.),

b Chi square test, (%)

**Table-II:** Role of complete blood cell count parameters in the demonstration of the success of single-dose methotrexate treatment.

Complete blood cell count parameters	Patients treated with single-dose methotrexate	Patients treated with surgery	P-value
NLR on Day 1	1.6582 (0.85–8.89)	2.1379 (1.12–5.17)	0.012
NLR on Day 4	1.5542 (0.71–13.33)	2.0000 (0.93–4.35)	0.032
NLR on Day 7	1.5247 (0.51–11.00)	1.9810 (0.57–6.67)	0.001
PLT on Day 1 (thousand mL)	236 (116–409)	219 (146–385)	0.151
PLT on Day 4 (thousand mL)	228 (72–434)	223 (142–337)	0.807
PLT on Day 7 (thousand mL)	232 (122–442)	222 (122–367)	0.566
PDW on Day 1 (%)	16.4 (13.6–18.7)	16.4 (15.5–17.4)	0.710
PDW on Day 4 (%)	16.4 (13.9–18)	16.5 (15.7–17.7)	0.794
PDW on Day 7 (%)	16.4 (13.9–18.7)	16.5 (16–17.8)	0.122

**Fig.1:**
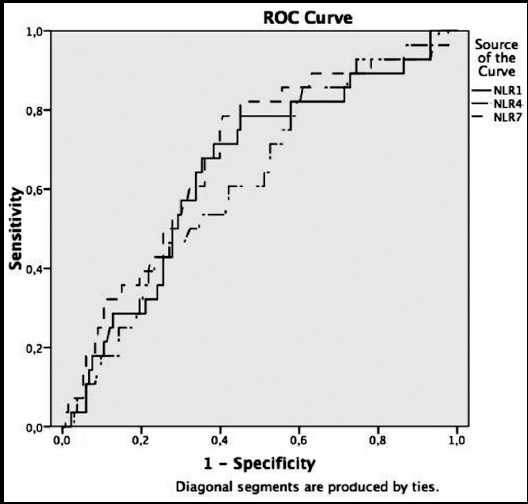
Relationship between NLR levels on Days 1, 4, and 7 and the demonstration of the success of single-dose methotrexate treatment.

Areas under the NLR roc curve on Days one, four and seven were found to be 0.654±0.058, 0.629±0.056, and 0.710±0.051, respectively, and the change in NLR on all three days was statistically significant (p=0.012, p=0.035, and p=0.001, respectively) (Table-III and Fig.1). The highest diagnostic test results between NLR on Days 1, 4, and 7 were detected for NLR on Day 7 (with 95% CI upper limit; sen 61.66%, spe 83.48%, positive predictive value 90.72%, and negative predictive value 25.97%) (Table-III).

**Table-III:** Univariate analyses of NLR levels on Days 1, 4 and 7.

	Area under curve	P-value	Cut-off value	Sensitivity	Specificity	Positive predictive value	Negative predictive value
NLR on Day 1	0.654±0.058	0.012	1.5626	38.06% (29.82%–46.84%)	48.15% (28.67%–68.05%)	83.53% (77.32%–88.29%)	17.11% (11.83%–24.10%)
NLR on Day 4	0.629±0.056	0.035	1.3944	35.07% (27.04%–43.79%)	62.96% (42.37%–80.60%)	83.93% (74.50%–89.33%)	17.14% (13.36%–21.73%)
NLR on Day 7	0.710±0.051	0.001	1.6043	53% (44.18%–61.66%)	66.67% (46.04%–83.48%)	83.61% (74.88%–90.72%)	17% (12.97%–25.97%)

## DISCUSSION

The objective of our study was to study the usage of complete blood cell count parameters in addition to the protocol with 15% BhCG decrease between Days 4 and 7, which is a frequently used method, for evaluating the success of single-dose methotrexate administration in this area.

The protocol with 15% decrease of BhCG between Days 4 and 7 that was recommended by Kirk E et al.^6^ in 2007 is still prevalently used as a protocol for the demonstration of success of single-dose methotrexate administration. In our study, in addition to monitoring BhCG levels, NLRs checked on Days 1, 4, and 7 following single-dose methotrexate administrations were found to be lower in the group with successful medical treatment.

Our results are consistent with those of certain studies in the literature.^9^,^14^ In the study conducted by Uysal G et al.^14^, it was found that NLR can be beneficial for single-dose methotrexate administration. In the study conducted by Turgut A et al.^9^, it was found that leukocyte values of the patient group with ectopic pregnancy rupture were significantly higher than that of the group without ectopic pregnancy rupture. Higher NLR values in the ectopic pregnancy group which required surgery can be explained by a prolonged inflammatory process.^13^ Studies in the literature on the role of NLR in demonstrating the success of single-dose methotrexate administration are limited. However, in the study by Karaman E et al. in which ectopic and intrauterine pregnancies were compared, but treatment success was not evaluated, it was found that there is no relationship between leukocyte count and ectopic pregnancy rupture.^15^ We believe that different NLR results are associated with the patient selection criteria and study design. To the best of knowledge, our study is the only study in literature to consecutively examine NLR values in addition to BhCG levels in ectopic pregnancy because of the tendency of inflammatory process to persist; therefore, comparing our results with those of other studies is difficult.

In the sub-analyses conducted with NLR levels on Days one, four and seven it was detected that NLR is sufficient in demonstrating the success of single-dose methotrexate treatment. However, in the diagnostic tests conducted with cut-off values determined using the roc curve, it was found that NLR levels are not as successful as the protocol with 15% decrease of BhCG between Days 4 and 7.

In some studies, it has been suggested that PDW and PLT, which are inflammatory markers, are associated with ectopic pregnancy, rupture, or surgical requirement in ectopic pregnancy.^9^,^10^,^16^ In our study, there was no statistically significant difference detected between the group with successful single-dose methotrexate treatment and the group with surgery requirement in terms of PDW percentage and PLT. Similarly, in the study conducted by Akkaya and Uysal,^14^ which was a rare study investigating whether there is a significant relationship between the success of single-dose methotrexate treatment and complete blood cell count parameters, no significant result was observed between surgery requirement and PLT and PDW percentage. We believe that the difference in our results were due to differences in the patient selection criteria and study design.

The strength of our study is that, to the best of our knowledge, it is the first known study in literature to establish a relationship between ectopic pregnancy and complete blood cell count parameters by examining consecutive values. In addition, it is one of the studies with the highest number of patients in literature. The limitation of our study was its retrospective design. However, limitations of a retrospective design were tried to be minimized by including patients with BhCG values <10 IU/L who were followed up in our hospital throughout this period.

## CONCLUSION

NLR, which is a complete blood cell count parameter, can be used for evaluating the success of single-dose methotrexate administration in treating ectopic pregnancy. However, it would be beneficial to conduct prospective randomized controlled studies consecutively examining complete blood cell count parameters for their role in the medical treatment of ectopic pregnancy to determine an adequate follow-up protocol as an alternative to monitoring BhCG levels.

### Authors’ Contribution

**AGK** conceived, designed and did statistical analysis & editing of manuscript.

**AHI** did data collection and manuscript writing.

**EB and AB** did review and final approval of manuscript.
